# Acknowledgement to Reviewers of *JCM* in 2017

**DOI:** 10.3390/jcm7010008

**Published:** 2018-01-09

**Authors:** JCM Editorial Office

**Affiliations:** MDPI AG, St. Alban-Anlage 66, 4052 Basel, Switzerland

Peer review is an essential part in the publication process, ensuring that *JCM* maintains high quality standards for its published papers. In 2017, a total of 116 papers were published in the journal. Thanks to the cooperation of our reviewers, the median time to first decision was 27 days and the median time to publication was 58 days. The editors would like to express their sincere gratitude to the following reviewers for their time and dedication in 2017:
Abenavoli, LudovicoLee, MargaretAguilera-Aguirre, LeopoldoLenaz, GiorgioAkita, SadanoriLi, Pin-LanAlbrecht, JanLimaye, Dnyanesh ArunAlexeyev, MikhailLindner, RobertAmato, PaulaLittman, DanAnderson, Amy E.Liu, Cheuk LunAndrès, EmmanuelLo, ChristopherAnsa, BenjaminLobb, ElizabethAnsquer, Jean ClaudeLong, AudreyAransay, Ana M.Lu, WeiliArdizzoni, ElisaLupien, AndréanneArnaout, BachaarLutz, GregoryArriens, CristinaLyons, Paul E.Athyros, VasiliosMagni, PaoloBachanova, VeronikaMaisch, B.Bader, PeterMalik, AfshanBaghdadi, Ziad D.Mansky, Kim C.Bajgoric, SanjinMarlicz, WojciechBar-Shavit, ZviMartin, FinianBarzilay, Joshua I.Martinez-Saguer, InmaculadaBatorova, AngelikaMayhan, William G.Bauer, NatalieMcAuley, Julie L.Beisswenger, ChristophMcCann, Fiona E.Benn, PeterMcCrae, NiallBernabeu, CarmeloMcGrath, RachelBernardy, NancyMcIntosh, E. David G.Bernat, James L.Mcmurray, David N.Berntorp, ErikMeads, Catherine A.Biagi, CarlottaMehrad, MitraBilal, JawadMeraj, Perwaiz M.Blatný, JanMessias, ErickBoateng, Joshua S.Minarovits, JanosBoccardo, FrancescoMizoguchi, ToshihideBonfield, Tracey L.Mogi, MasakiBooth, Stephanie A.Morell, FerranBoulware, David R.Morello, RoyBowler, SimonMorgan, Michael A. M.Branchini, AlessioMorgan-Warren, Peter J.Bratton, Susan L.Mortiboys, HeatherBravaccio, CarmelaMosna, FedericoBricout, MarineMoutou, CelineBrumeanu, Teodor-DoruNarita, NorihikoBuechler, ChristaNatarajan, Sathish KumarBugiardini, EnricoNestler, UlfBurger, Charles D.Neureiter, DanielBurtey, StéphaneNguyen, T. K. P.Calhelha, Ricardo C.Niyazov, Dmitriy M.Camire, Rodney M.Nogami, KeijiCarcao, ManuelNolan, Vikki G.Chase, ChristopherNorth, CarolChen, Yung-HsiangObermüller, NicholasChiavaroli, LauraPage, Anne-LaureChiloiro, GiudittaPalaniappan, BalasubramanianCho, Mi-LaPalla, RobertaChristiansen, Christian F.Pantazis, AntonisCiccone, Marco MatteoPaquis-Flucklinger, VéroniqueCicero, ArrigoParolini, CinziaClarke, KieranPatel, IndravadanClaydon, Leica SarahPedraza-Chaverri, JoséCooper, Robert G.Perarnau, Jean MarcCortesi, EnricoPérez-Botero, JulianaCsiszar, AgnesPerricone, CarloDaehn, IlsePeterson, Eliza J. R.Dallenga, TobiasPfeffer, GeraldDallner, GustavPicca, StefanoDavos, ConstantinosPointer, Mildred A.De La Rosa Carrillo, DavidPokorn, MarkoDe Paepe, BoelPollock, Richard L.De Paiva, Cintia S.Pozzilli, PaoloDe Rivera, JosephPreissner, Klaus T.DeBeer, Bryann B.Principe, Maria Ilaria DelD'elios, Mario M.Ramani, VijayDetweiler, Mark B.Randell, Scott H.DiMauro, SalvatoreRauch, BernhardDiMeglio, Linda A.Raychaudhuri, Siba P.Dimopoulos, KonstantinosRibera, José MariaDoi, TakehikoRice, JohnDu, HuiyunRingström, GiselaDusi, VeronicaRo, EunyoeEiges, RachelRobins, Harlan S.Eiroa-Orosa, Francisco JoseRodeghiero, FrancescoEisenbarth, HedwigRodenburg, RichardEljaafari, AssiaRodriguez-Merchan, Emerito CarlosEsteso Díez, Milagros CristinaRoychowdhury, SanjoyExadaktylos, AristomenisRusnati, MarcoFadda, RobertaSakata, KiyohikoFavaloro, EmmanuelSala, AngeloFennell, KateSalek, SherveenFeola, MauroSaner, Fuat HakanFilosto, MassimilianoSantulli, GaetanoFlores-Montero, JuanSatake, HironagaFranco, LuisSawa, TeijiFruchtman, YarivSchmid, Jean-PaulGadjeva, MihaelaSchmitt, Ulrich R.Gajula, RajendraSeidman, LauraGarcia-Redondo, Ana BelenSen, SouvikGebhart, JohannaSenbonmatsu, TakaakiGeurts, Jose W.Shankar, EswarGiacomin, PaulShanmuganathan, NaranieGitelman, Stephen E.Sheehan, VivienGłąbska, DominikaSherbet, Gajanan V.Greenberg, ArthurSherwin, LeeAnne B.Gris, Jean ChristopheSié, PierreHargreaves, IainSimecka, Jerry W.Hatachi, YukimasaSimpson, David A. C.Heales, SimonSingh, SeemaHeitink-Pollé, Katja M. J.Skapenko, AllaHelmby, HelenaSmith, KeriHendren, RobertSoiza, Roy L.Hohenberger, GloriaSoucie, MikeHolme, Pål AndreSpagnuolo, RoccoHorgan, GrahamStanton, Robert C.Horton, MarkStenmark, KurtHorvath, RitaStevens, Natalie R.Hourigan, ChristopherSugiyama, TakashiHouston, Mark C.Sukocheva, OlgaIlatovskaya, DariaSukumaran, AnilIlluminati, GiulioSuzuki, NoboruIm, Sin-HyeogSvendsen, Morten Bo SøndergaardImamura, TeruhikoSwerdlow, RussellIshibashi, KenichiSzablewski, LeszekIshii, TetsuyaSzolnoky, GyőzőJeanmart, StephaneTagarakis, GeorgiosJensen, KatrinTakayama, YoshiharuJimenez-Rondan, FelixTaube, JoeJin, ZhongminTekle, MichaelJohnson, Martin K.Teotia, PoojaJoshi, Pushpa RajTheilmann, LorenzJowett, AdamThoene, JessKaaja, RistoThoma, MyriamKadlecek, StephenThornton, DavidKarras, SpirosTiano, LucaKathrins, MartinTonolo, GiancarloKawanami, DaijiTrebicka, JonelKeipert, ChristineTsoulfas, GeorgeKneepkens, C. M. FrankTuck, CarolineKofler, MarkusTulloh, RobertKojima, MasamiVan der Velden, Vincent H. J.Kok, Victor C.Van Dyke, KnoxKotake, ShigeruVan Ryn, JoanneKotloski, RobertVandergheynst, FrédéricKozela, EwaVaretto, GianfrancoKrysinska, KarolinaVork, LisaKubota, YoshiakiVyas, JulianKumar, GauravWada, JunKumar, SajeeshWang, QiangKunz, WolframWang, Tsung-JenKurasawa, KazuhiroWang, YibinKurdowska, Anna K.Watson, Paul J.Kuzmiene, LoretaWeir, GordonKwakowsky, AndreaWilliams, Joah L.La Rocca, Renato V.Winkler, JohannesLadetto, MarcoWu, Wen-ShengLadjemi, Maha ZohraWydau, SandraLanz, Tobias V.Yadollahpour, AliLarsen, S.Yamakawa, HideakiLaubach, Victor E.Yin, JunLaurent, Louise C.Ying, Gui-shuangLee, Kyung-YilZaheer, Asgar

